# Case report: The cardio-facio-cutaneous syndrome due to a novel germline mutation in *MAP2K1*: A multifaceted disease with immunodeficiency and short stature

**DOI:** 10.3389/fped.2022.990111

**Published:** 2022-10-14

**Authors:** Aleksandra Szczawińska-Popłonyk, Natalia Popłonyk, Marek Niedziela, Anna Sowińska-Seidler, Paweł Sztromwasser, Aleksander Jamsheer, Monika Obara-Moszyńska

**Affiliations:** ^1^ Department of Pediatric Pneumonology, Allergy and Clinical Immunology, Institute of Pediatrics, Poznań University of Medical Sciences, Poznań, Poland; ^2^ Student Scientific Society for Pediatric Endocrinology, Poznań University of Medical Sciences, Poznań, Poland; ^3^ Department of Pediatric Endocrinology and Rheumatology, Institute of Pediatrics, Poznań University of Medical Sciences, Poznań, Poland; ^4^ Department of Medical Genetics, Poznań University of Medical Sciences, Poznań, Poland; ^5^ Department of Biostatistics and Translational Medicine, Medical University of Łódź, Łódź, Poland; ^6^ MNM Diagnostics, Poznań, Poland

**Keywords:** MAP2K1, rasopathy, immunodeficiency, short stature, craniofacial dysmorphism, immunoglobulins, growth hormone

## Abstract

Cardio-facio-cutaneous syndrome (CFCS) belongs to the group of RASopathies, clinical disorders defined by disruptions in the RAS/MAPK signaling pathway. It is caused by heterozygous gain-of-function germline mutations in genes encoding protein kinases: *BRAF*, *MAP2K1* (*MEK1*), *MAP2K2* (*MEK2*), and in the GTPase-encoding gene *KRAS*. CFCS is characterized by craniofacial dysmorphic features, congenital heart defects, severe malnutrition, proportionate short stature, anomalies within the structure of skin and hair, and psychomotor disability. The pathophysiology of growth impairment is multifactorial with feeding difficulties, growth hormone deficiency, and insensitivity. Immunodeficiency has not been hitherto reported as an integral part of CFCS yet an increased activation of the RAS/MAPK signaling pathway may contribute to explaining the causal relationship between RASopathy and the dysfunctions within the B and T lymph cell compartments resulting in a deficiency in T cell costimulation and B cell maturation with impaired class switch recombination, somatic hypermutation, and high-affinity antibody production. We report on a boy born prematurely at 32 WGA, with the perinatal period complicated by pneumonia, respiratory distress syndrome, and valvular pulmonary stenosis. The boy suffered from recurrent pneumonia, obstructive bronchitis, sepsis, urinary tract infection, and recurrent fevers. He presented with severe hypotrophy, psychomotor disability, short stature, craniofacial dysmorphism, dental hypoplasia, sparse hair, and cryptorchidism. Whole genome sequencing showed a novel heterozygous pathogenic germline missense variant: c.364A > G; p.Asn122Asp in the *MAP2K1* gene, supporting the diagnosis of CFCS. The immunological workup revealed hypogammaglobulinemia, IgG subclass, and specific antibody deficiency accompanied by decreased numbers of T helper cells and naive and memory B cells. Replacement immunoglobulin therapy with timely antibiotic prophylaxis were instituted. At the age of six years, growth hormone deficiency was diagnosed and the rGH therapy was started. The ever-increasing progress in genetic studies contributes to establishing the definitive CFCS diagnosis and sheds the light on the interrelated genotype-phenotype heterogeneity of RASopathies. Herein, we add new phenotypic features of predominating humoral immunodeficiency to the symptomatology of CFCS with a novel mutation in *MAP2K1.* While CFCS is a multifaceted disease, increased pediatricians’ awareness is needed to prevent the delay in diagnostics and therapeutic interventions.

## Introduction

*RAS* genes constitute a multigene family including *HRAS, NRAS,* and *KRAS* encoding a group of small guanosine nucleotide-bound GTPases that act as an essential cellular signaling axis. These RAS-GTPases control activation of the downstream RAF-MEK-ERK pathway, constituting the mitogen-associated protein kinase (MAPK) cascade that is vital for a multiplicity of cellular processes in the nucleus and cytosol, including survival, proliferation, differentiation, motility, and apoptosis. They are activated by various extracellular stimuli, such as growth factors binding to receptor tyrosine kinase, cytokine receptors, and extracellular matrix receptors. Subsequently, activated RAS phosphorylate downstream transducers: RAF proteins (ARAF, BRAF, and CRAF), MEK1 and/or MEK2, and finally, ERK1 and/or ERK2 (1, 2). The complex nature of the RAS/MAPK signaling pathway with the multiplicity of mechanisms cumulated in the RAS/MAPK pathway dysregulated activations as their common pathogenetic denominator.

Cardio-facio-cutaneous syndrome (CFCS) belongs to the group of the RASopathies, a spectrum of clinical disorders caused by germline mutations in components or regulators of the RAS/MAPK pathway. With its numerous genes and overlapping regulatory mechanisms involved, interfering with other cellular pathways contribute to the complex genotype-phenotype correlations and the heterogeneity of phenotypic features (3, 4). This pathway-based, mechanistic approach to defining RASopathies makes these medical genetic syndromes unique as opposed to the isolated one gene-one syndrome approach (5–7). In CFCS, heterozygous gain-of-function mutations occur in genes encoding protein kinases: *BRAF*, accounting for 75% of the genetic background in the syndrome, *MAP2K1* (*MEK1*), *MAP2K2* (*MEK2*), both found in 25% of the mutation-positive patients, and in the GTPase-encoding gene *KRAS*, constituting the rarest genetic background, found in less than 2% of the CFC patients (5, 8). An activating YWHAZ variant in the RAF-ERK pathway has also been reported in individuals with clinical syndromic features consistent with CFCS thereby expanding the spectrum of deleterious gain-of-function mutations underlying the characteristic phenotype (9). Recently, 19p13.3 microdeletion including the *MAP2K2* gene in a newborn patient with CFCS and severe clinical phenotype has also been reported (10). Consequently, CFCS is a phenotypically heterogeneous disorder characterized by craniofacial dysmorphic features, congenital heart defects, severe malnutrition, proportionate short stature, anomalies within the structure of skin and hair, and psychomotor disability (5, 8, 9, 11, 12).

In a mathematical multifactorial correlation study, a degree of associations of clinical traits and their frequencies were verified to calculate the CFC index thereby proposing an objective method for the easier recognition and more accurate clinical diagnosis of CFCS (13). It is worth noting that the highest index has been attributed to developmental disability, neurocognitive and motor difficulties (14, 15), craniofacial dysmorphic features such as high cranial vault, macrocephaly, bitemporal narrowing, depressed nasal bridge, anteverted nostrils (16, 17), dysmorphologic, sparse hair (18), hyperkeratotic skin (19), as well as congenital heart defect and hypertrophic cardiomyopathy (20–22) and short stature (23, 24). The pathophysiology of growth impairment is multifactorial with feeding difficulties, growth hormone (GH) deficiency, and insensitivity have been postulated as possible contributors to short stature.

Finally, clinical manifestations of CFCS in individual patients may overlap and result from heterogeneous pathological mechanisms governing the ultimate genotype-phenotype relationship. Wide symptomatology associated with inflammatory and autoimmune disorders (25), energy metabolism disturbances, myeloproliferative disease (26), and tumorigenesis (27) may be, therefore, observable in affected patients at different stages of their development (28–30). Immunodeficiency associated with syndromic features has not been hitherto thoroughly studied and frequently reported as an integral part of CFCS (31), yet an increased activation of the RAS/MAPK signaling pathway may contribute to explaining the causal relationship between RASopathy and the regulation of immune cells development and functions. It may be therefore assumed that dysregulation in cellular processes within the B and T lymphocyte compartments may result in deficiency in T cell costimulation and B cell dysfunctions with impaired class switch recombination (CSR), somatic hypermutation (SHM), and high-affinity antibody production.

## Case presentation

### The patient

**Figure 1:**
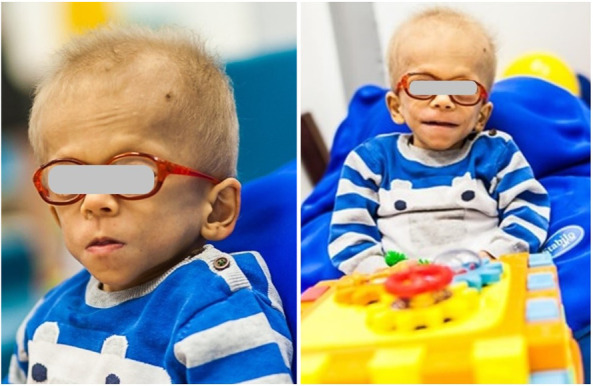
Phenotypical features of the patient with CFCS type 3 due to a novel pathogenic germline missense variant (NM_002755.3:[c.364A > G; p.Asn122Asp) in the *MAP2K1* gene.

**Figure 2:**
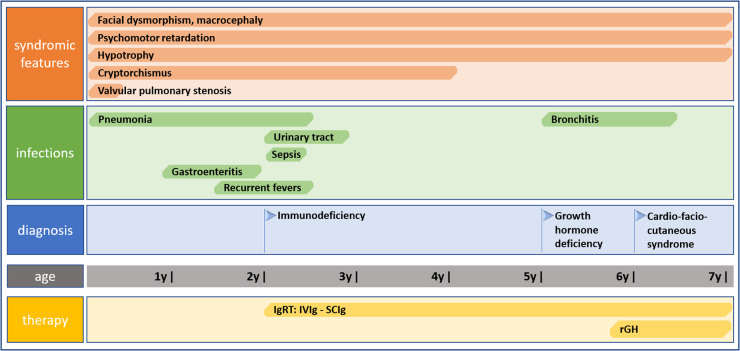
Timeline showing clinical case history including symptomatology, infectious history, diagnosis of CFCS, and therapy.

We report on a case of a boy who was referred to our pediatric clinical hospital at the age of two years due to recurrent respiratory tract infections for immunodiagnostics. His antenatal history was remarkable for polyhydramnios and supported pregnancy due to the signs of life threat to the fetus since the 26 week gestational age (WGA). Due to polyhydramnios, repeated amniocentesis and drainage of the amniotic fluid were performed, and chorioamnionitis was an indication to terminate the pregnancy. He was born prematurely by cesarean section at 32 WGA, and the perinatal period was complicated by pneumonia, respiratory distress syndrome, and cardiac insufficiency due to valvular pulmonary stenosis. Since birth, he required combined antibiotic therapy and mechanical ventilation in the neonatal intensive care unit because of respiratory and circulatory insufficiency. He also presented with craniofacial dysmorphism and cryptorchidism raising the suspicion of Noonan syndrome yet sequencing of the *PTPN11* gene did not show any mutation. By the age of two years, the boy suffered from recurrent pneumonia and bronchitis, staphylococcal sepsis, urinary tract infection, and recurrent fevers with *Staphylococcus aureus* and *Pseudomonas aeruginosa* repeatedly cultured in tracheal aspirates. Since the age of four months, during the first two years of life, the number of pneumonia episodes was four every year, severe enough to require hospitalizations. In the first year of life, he underwent a multistep corrective surgery of valvular pulmonary stenosis. Due to the failure of thrive and feeding difficulties, gastrostomy was created to improve his nutritional status, yet poor weight gaining and recurrent fevers prompted the surgical team to remove it. The patient received a live Bacillus Calmette-Guerin (BCG) vaccine after birth and a measles-mumps-rubella (MMR) trivalent vaccine at the age of 13 months was not recommended due to recurrent infections. Inactive vaccines were administered without adverse effects and the diphtheria, tetanus, and acellular pertussis (DTaP) booster dose was given at the age of six years, 10 weeks before the immunological workup. On admission to our pediatric immunology unit, he presented with severe hypotrophy, psychomotor retardation, short stature, macrocephaly, facial dysmorphism with prominent forehead, depressed nasal bridge, and anteverted nostrils, macrostomia, dental hypoplasia, low-set ears, sparse hair with absent eyebrows and eyelashes, and bilateral cryptorchidism. The patient’s phenotypic features are displayed in Figure 1. In-depth genetic studies including whole genome sequencing (WGS) showed a novel heterozygous pathogenic germline missense variant (NM_002755.3:[c.364A > G; p.Asn122Asp) in the *MAP2K1* gene and the diagnosis of the cardio-facio-cutaneous syndrome (OMIM #615279) was established. The detected missense substitution resulted in a change of asparagine into aspartic acid in a highly conserved amino acid residue 122. Furthermore, the variant occurred *de novo* as it has been shown in targeted parental studies. To follow the case history, see Figure 2 (Timeline). The immunological workup revealed hypogammaglobulinemia, IgG subclass, and specific antibody deficiency accompanied by a decreased numbers of T helper cells and abnormalities within the B cell compartment with low numbers of naive and switched memory B cells (Data shown in Table 1). Replacement immunoglobulin therapy (IgRT) with intravenous (IVIg) followed by subcutaneous immunoglobulins (SCIg) along with timely antibiotic prophylaxis were instituted leading to significant improvement and reducing the infections rate. The regular IgRT has led to a remarkable alleviation of respiratory symptoms, and since the age of three years, he suffered from two episodes of bronchitis and episodic mild upper airway infections and, aged six years, required an admission to the hospital.

**Table 1:** The immunological workup with antibody-mediated response and peripheral blood flow cytometric immunophenotyping in the CFCS patient.

Immunological workup	Age 2 years (1)	Reference values (1)	Age 6 years (2)	Reference values (2)
**Antibody response**				
**Immunoglobulins**				
IgG	184 mg/dl	520–1360 mg/dl	265 mg/dl	570–1410 mg/dl
IgA	19 mg/dl	45–135 mg/dl	73 mg/dl	65–240 mg/dl
IgM	28 mg/dl	46–190 mg/dl	5 mg/dl	55–210 mg/dl
**IgG subclasses**				
IgG1	95 mg/dl	315–945 mg/dl	193 mg/dl	306–945 mg/dl
IgG2	41 mg/dl	36–225 mg/dl	124 mg/dl	60–345 mg/dl
IgG3	7 mg/dl	17–68 mg/dl	18 mg/dl	99–122 mg/dl
IgG4	0.5 mg/dl	1–54 mg/dl	2 mg/dl	20–112 mg/dl
**Antigen-specific antibodies**				
Anti-diphtheria toxoid			<0.1 IU/ml	>1 IU/ml
Anti-tetanus toxoid			<0.1 IU/m	>1 IU/ml
**PB lymphocyte immunophenotyping**				
WBC	10210 cc		14020 cc	
Lymphocytes CD45+/SSC low	36.0%, 3676 cc	29.6–69.2%, 2300–6900 cc	28.0%, 3856 cc	29.6%–49.8%, 1700–3600 cc
B CD19+	11.0%, 209 cc	14.1–28.5,0%, 400–1700 cc	4.0%, 178 cc	9.7%–23.7%, 300–600 cc
Transitional B CD19 + CD38 + IgM++	23.7%, 99 cc	3.1%–12.3%, 20–200 cc	1.6%, 3 cc	4.6%–8.3%, 10–40 cc
Mature naїve B CD19 + CD27-IgD+	94,8%, 395 cc	54.0%–88.4%, 280–1330 cc	64.4%, 115 cc	47.3%–77.0%, 130–460 cc
Non-switched memory B (MZL) CD19 + CD27 + IgD+	2.0%, 4cc	2.7%–19.8%, 20–180 cc	12.3%, 22 cc	5.2%–20.4%, 20–100 cc
Switched memory B CD19 + CD27 + IgD-	2.1%, 5 cc	4.7%–21.2%, 20–220 cc	2.3%, 4 cc	10.9%–30.4%, 40–140 cc
Immature B CD19 + CD21lo	9.1%, 19 cc	4.1%–24.4%, 20–230 cc	4.9%, 9 cc	5.9%–25.8%, 20–120 cc
Activated B CD19 + CD38loCD21lo	4,6%, 11 cc	1.7%–5.4%, 10–60 cc	4.9%, 9 cc	2.3%–10.0%, 10–40 cc
Plasmablasts CD19 + CD38++IgM-	0.0%, 0 cc	0.6%–4.0%, 5–10 cc	0.0%, 0 cc	0.6%–5.3%, 0–3 cc
T CD3+	74.0%, 1491 cc	52.0%–92.0%, 850–4300 cc	47.0%, 1905 cc	55.0%–97.0%, 850–4300 cc
T helper CD3 + CD4+	34.0%, 1288 cc	25.0%–66.0%, 500–2700 cc	18.0%, 722 cc	26.0%–61.0%, 500–2700 cc
T suppressor/cytotoxic CD3 + CD8+	19.0%, 720 cc	9.0%–49.0%, 200–1800 cc	23.0%, 916 cc	13.0%–47.0%, 200–1800 cc
CD4+/CD8+	1.79	1.5–2.5	0.79	1.5–2.5
Recent thymic emigrants CD3 + CD4 + CD45RA + CD31+	47.0%, 607 cc	37,0%–100%, 190–2600 cc	33.8%, 244 cc	41.0%–81.0%, 190–2600 cc
Naïve T helper CD3 + CD4 + CD45RA + CD27+	66.1%, 851 cc	52.0%–92.0%, 300–2300 cc	47.9%, 345 cc	46.0%–99.0%, 300–2300 cc
Central memory T helper CD3 + CD4 + CD45RA-CD27+	2.9%, 307 cc	15.0%–56.0%, 160–660 cc	45.3% 327 cc	0.35%–100%, 160–660 cc
Effector memory T helper CD3 + CD4 + CD45RA-CD27-	9.6%, 123 cc	0.3%–9.0%, 3–89 cc	5.9%, 42 cc	0.3%–18.0%, 3–89 cc
Terminally differentiated memory T helper CD3 + CD4 + CD45RA + CD27-	0.4%, 5 cc	0.0%–1.2%, 0–16 cc	1.0%, 7 cc	0.0%–1,8%, 0–16 cc
Follicular CXCR5+ T helper CD3 + CD4 + CD45RO + CD185+	16.0%, 206 cc	6.0%–72.0%, 13–170 cc	11.2%, 29 cc	7.0%–85.0%, 13–170 cc
Regulatory T helper CD3 + CD4 + CD25++CD127-	0.5%, 6 cc	3.0%–17.0%, 39–150 cc	6.8%, 49 cc	4.0%–14.0%, 39–150 cc
Naïve T suppressor/cytotoxic CD3 + CD8 + CD27 + CD197+	27.7%, 199 cc	19.0%–100%, 53–1100 cc	26.1%, 239 cc	16.0%–100%, 53–1100 cc
Central memory T suppressor/cytotoxic CD3 + CD8 + CD45RA-CD27 + CD197+	1.2%, 8 cc	1.0%–9.0%, 4–64 cc	2.2%, 20 cc	1.0%–6.0%, 4–64 cc
Effector memory T suppressor/cytotoxic CD3 + CD8 + CD45RA-CD27-CD197-	7.4%, 53 cc	10.0%–55.0%, 24–590 cc	19.0%, 174 cc	5.0%–100%, 24–590 cc
Terminally differentiated T suppressor/cytotoxic CD3 + CD8 + CD45RA + CD27-CD197-	3.1%, 22 cc	6.0%–83.0%, 25–530 cc	12.8%, 117 cc	15.0%–41.0%, 25–530 cc
NK CD3-CD45 + CD16 + CD56+	22.0%, 808 cc	2%–25%, 61–510 cc	19.0%, 754 cc	2.0%–25.0%, 61–510 cc

At the age of five years, the boy was referred to the department of pediatric endocrinology for hormonal assessment. On admission, his height was 86 cm corresponding with the standard deviation score (SDS) −8.3 and the body mass index (BMI) was corresponding with SDS −6.1. The evaluation of the endocrine system showed partial growth hormone deficiency with remarkably low insulin growth factor 1 (IGF-1) concentration, and the bone age was estimated at 1.5 years. The recombinant growth hormone (rGH) therapy was started at the age of 6.5 years, reaching the height of −7.0 SDS, height velocity of 7 cm/year, the bone age of 4.5 years, and increasing the IGF-1 level after one year of the therapy. Data including hormonal laboratory parameters and a growth chart are displayed in Table 2.

**Table 2:** Endocrinological workup of the presented CFCS patients before and one year after starting the rGH therapy, aged 6 and 7 years old, respectively.

Hormonal Parameters	Before starting rGH	One year after starting rGH	Reference values	Growth chart
Max GH after onset of sleep	7.1 ng/ml		>10.0 ng/ml	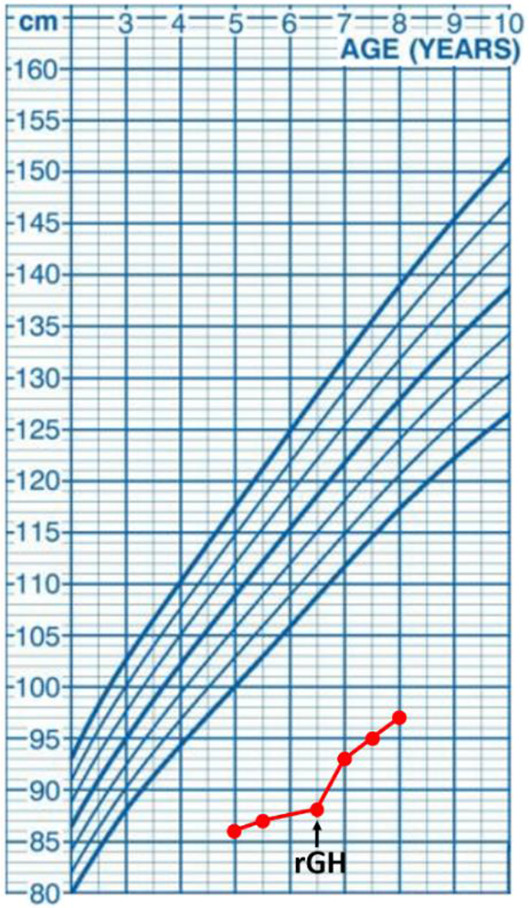
Max GH after glucagon	9.4 ng/ml		>10.0 ng/ml	
Max GH after clonidine	7.2 ng/ml		>10.0 ng/ml	
IGF-1	12.0 ng/ml	90.0 ng/ml	115.0–249.0 ng/ml	
IGFBP-3	838.0 ng/ml	2462.0 ng/ml	2846.0–4462.0 ng/ml	
LH	0.2 mIU/ml		0.02–1.03 mIU/ml	
FSH	0.5 mIU/ml		0.25–1.92 mIU/ml	
Testosterone	0.45 nmol/L		0.1–1.0 nmol/L	
PRL	86.58 ng/ml		4.79–23.3 ng/ml	
PRL after precipitation with PEG	27.52 ng/ml		4.79–23.3 ng/ml	
Cortisol at 8:00 am	187.0 ng/ml		37.0–194.0 ng/ml	
Cortisol after glucagon	189.0 ng/ml		>180.0 ng/ml	
ACTH	109.6 pg/ml		10.0–60.0 pg/ml	
TSH	3.993 µIU/ml		0.700–4.170 µIU/ml	
FT3	1.54 pg/ml		0.86–1.37 pg/ml	
FT4	3.77 ng/dl		2.79–4.42 ng/dl	
anti-TPO Ab	7.9 IU/ml		<5.61 IU/ml	
anti-TG Ab	1.9 IU/ml		<4.11 IU/ml	
Insulin	2.2 µU/ml		<15.0 µU/ml	
PTH	57.6 pg/ml		10.0–60.0 pg/ml	
25-OH-D	36.8 ng/ml		30.0–50.0 ng/ml	

## Diagnostic assessment

### Whole genome sequencing

Genomic DNA was extracted from peripheral blood (PB) leukocytes according to standard procedures. The sequencing library was prepared by Macrogen Inc. (Seul, Korea) using TruSeq DNA PCR-free kit (Illumina Inc, San Diego, California, USA) and 550 bp inserts. The library was sequenced on the Illumina Novaseq 6,000 platform using 150 bp paired-end reads following standard protocols. Bioinformatic analysis was performed as previously described (32). FastQC was used to confirm the quality of the sequenced reads which were mapped to the human reference genome GRCh38 using Speedseq framework v.0.1.2 (BWA MEM 0.7.10, Sambamba v0.5.9). Mapping coverage was calculated using Mosdepth 0.2.4. Sequence variants were detected using DeepVariant 0.8.0. and CNVnator v0.4, and annotated using Ensembl Variant Effect Predictor 97.3 (VEP). Variants with minor allele frequency below 0.5% or missing from gnomAD v3 database, and missing from an inhouse database of over 1,200 ethnically matched WGS samples (33) were selected for the analysis; variants in genes associated with RASopathies and immunodeficiency were prioritized. Variants in genes associated with immunodeficiency were reanalyzed using the minor allele frequency threshold below or equal to 3%. The clinical interpretation of detected mutations was performed based on various online databases of genomic variants including ClinVar2, GnomAD3, Human Gene Mutation Database (HGMD) Professional 2014.1. The pathogenicity of the identified variants was evaluated by multiple prediction tools integrated into WGS data analysis pipeline and VarSome Premium variant data analysis tool (34). The classification of the reported mutation was performed according to the American College of Medical Genetics and Genomics and the Association for Molecular Pathology (ACMG/AMP) (35). The *de novo* occurrence of the pathogenic mutation in the proband was confirmed in parental testing employing targeted Sanger sequencing. A detailed summary of the pathogenicity prediction of the detected *MAP2K1* mutation including pathogenicity and conservation scores is shown in Supplementary Table S2.

### Flow cytometric peripheral blood lymph cell immunophenotyping

Cells were labelled with the following murine fluorochrome-stained monoclonal antibodies: anti-CD45 FITC (fluorescein isothiocyanate), anti-CD14 PE (phycoerythrin), anti-CD19 PE, anti-CD19 PerCP (peridinin chlorophyll protein), anti-IgM FITC, anti-IgD FITC, anti-CD38 APC (allophycocyanin), anti-CD27 PE, anti-CD21 FITC, as well as anti-CD3 FITC, anti-CD4 FITC, CD45RA FITC, CD127 FITC, CD185 FITC, anti-CD8 PE, anti-CD16 + CD56 PE, CD25 PE, CD31 PE, CD45RO PE, anti-CD3 PerCP, CD197 PerCP, anti-CD4 APC and anti-CD8 APC (all Beckton-Dickinson Biosciences, USA). The acquisition of cells and analysis was carried out with the use of the flow cytometer FACSCanto and FACSDiva software (Beckton-Dickinson, USA). With sequential gating on biparametric scattering CD45 + CD14- lymphocytes, the following lymphocyte subpopulations were identified:

– CD19+ B cells, immature CD19 + CD21lo, immature activated CD19 + CD38loCD21lo, transitional CD19 + CD38hisIgMhi, non-switched memory CD19 + CD27 + sIgD+, switched memory CD19 + CD27 + IgD- B cells, and CD19 + CD38hisIgM- plasmablasts
– CD3+ T cells, CD3 + CD4+ T helper cells, CD3 + CD4 + CD31 + CD45RA+ recent thymic emigrants, naïve CD3 + CD4 + CD27 + CD45RA+, regulatory CD3 + CD4 + CD25++CD27-, central memory CD3 + CD4 + CD27 + CD45RO+, effector memory CD3 + CD4 + CD27-CD45RO+, terminally differentiated CD3 + CD4 + CD27-CD45RA+, follicular CD3 + CD4 + CD185 + CD45RO+, and regulatory CD3 + CD4 + CD45RO + CD127-CD25++ T helper cells. Among CD3 + CD8 + cytotoxic T cells, the following subsets were distinguished: naïve CD3 + CD8 + CD197 + CD27 + CD45RA+, central memory CD3 + CD8 + CD197 + CD27 + CD45RO+, effector memory CD3 + CD8 + CD197-CD27-CD45RO+, and terminally differentiated CD3 + CD8 + CD197-CD27-CD45RA+ cells.
– CD3-CD16 + CD56+ NK cells.

The relative values of PB lymphocytes, the B, T, and NK cells of the total lymphocyte population as well as B and T cell subsets were calculated. The absolute counts of all cell subsets were calculated from the PB leukocyte counts. A comparative analysis was done with the reference cut-off values of B (36) and T cell subsets (37) for pediatric populations at different age groups.

### Auxology

The boys’ height was measured in the lying position due to cerebral palsy using SECA measuring mat. The height and BMI SDS for chronological age were calculated using WHO references, and the bone age was estimated according to the Greulich and Pyle method for evaluation of the skeletal developments of the hand and wrist.

## Discussion

Addressing the concerns on the heterogeneity of clinical features, the rarity of the syndrome, and complex genotype-phenotype relationships, it needs to be highlighted that the definitive diagnosis of CFCS is challenging for clinicians (38). Attempts have been made to better delineate the phenotypic perinatal (39) and childhood presentation (13) to facilitate the early diagnosis. Functional consequences of mutations in the CFCS-related RAS/MAPK pathway involving KRAS-BRAF-MAPK-ERK components show clinical variety as well, making the interrelated links between the causative pathogenic variant and the patient’s phenotype difficult to predict. RAS/MAPK pathway plays pleiotropic roles at the crossroads of the development and homeostasis of endocrine and metabolic tissues. Directing the metabolism towards anabolic processes, such as macropinocytosis and autophagy as well as regulating the response to foods through neuroendocrine signals, the RAS/MAPK pathway acts as a modifier of the hormonal and metabolic balance (40) as well as bioenergetics related to mitochondrial physiology and high energy expenditure (41). The response to hormones, such as insulin, leptin, and GH, as well as the development of hormonally active organs, such as the hypothalamus, pancreas, and adipose tissue has been associated with the activation of the RAS/MAPK cascade.

Importantly, the RAS/MAPK pathway is mobilized downstream from the GH receptor and in RASopathies, increased activation of the RAS/MAPK signaling results in reduced IGF-1 generation in response to GH (39). The most widely proposed hypothesis of growth failure is a partial GH insensitivity due to a post-receptor signaling defect (42, 43). Nonetheless, the pathomechanism of short stature in RASopathies is complex and multifactorial, and besides GH deficiency, partial GH insensitivity, neurosecretory dysfunction, also neuromuscular, orodental and feeding disorders, and history of cardiac surgery have been proposed as contributory disorders (42–44). In our patient, severe hypotrophy, poor nutritional status, feeding disorders, muscular atrophy and hypotonia, as well as a history of valvular pulmonary stenosis in parallel with recurrent infections had a salient effect on his developmental impairment and growth failure. Moreover, failure to thrive may underpin immunodeficiency and, in turn, immunodeficiency may escalate failure to thrive and hormonal dysfunction, creating a vicious circle in pathomechanisms of immunity and development. In RASopathies, an increased RAS/MAPK pathway signaling in chondrocytes may impair growth plate development and longitudinal growth (45). MAPK activation is important in regulating the proliferation of pituitary somatotrophs and, therefore, proper GH secretion (46). The partial GH insensitivity in RASopathies has also been postulated, implying that the response to rGH in MAP2K1 deficiency-related CFCS may not be entirely satisfactory (42, 43, 47). In the presented patient, the GH secretion was just below the cut-off value and was accompanied by a very low IGF-1 and IGF-binding protein 3 (IGFBP-3) that might suggest a coexisting GH insensitivity. Interestingly, the elevated prolactin (PRL) concentration with nearly normal precipitation with polyethylene glycol (PEG) may be hypothesized to result from immunological disturbances and IgRT in the patient.

The pleiotropic effect of RAS-associated pathways on cellular growth, differentiation, and apoptosis may also be hypothesized as a potential background for the combined immunodeficiency in the patient studied. The Ras/MAPK cascade cumulating in ERK kinase underlie functional switching in lymph cells. Engagement of antigen receptors in lymph cells stimulates Ras proteins activation by guanine nucleotide exchange factors (GEFs): RasGRP1 acts downstream from antigen receptors in T cells, whereas RasGRP1 and RasGRP3 function in B cells (48–52). Biallelic loss-of-function (LOF) mutations in the *RASGRP1* gene have been described in several patients to develop a combined immunodeficiency (CID) and impaired cytoskeleton dynamics, susceptibility to severe viral, fungal, and bacterial infections, autoimmune cytopenias, and an Epstein-Barr virus (EBV)-driven lymphoproliferation. The immunodeficiency in RASGRP1 is characterized by impaired B and T cell activation and proliferation, decreased T cell numbers, and NK cell cytotoxic dysfunction (53). It has also been shown that in B cells, positive feedback-driven Ras activation is the proposed source of digital MAPK responses and signal amplification following antigen stimulation at the B cell receptor (54). Whereas the regulatory role of MAPK has been shown in crucial cellular processes, including driving proliferation and activation of dendritic cells, it has been hypothesized that the MAPK cascade promotes efficient adaptive immune response (55). While the *MAP2K1* variants have also been shown to activate the ERK-dependent cell cycle progression and autophagy (56), it has raised the question whether the autophagy-mediated altered MAP2K1 function contributes to a dysfunctional immunophenotype. Referring to the two hitherto reported CFCS patients with hypogammaglobulinemia and absent antigen-specific antibody response, both harbored the same c.389A > G; p.Tyr130Cys mutation in the *MAP2K1* gene (31). While in our patient, developmental disorders within the lymphocyte compartment have been found, it raises questions regarding the role of this novel pathogenic c.364A > G; p.Asn122Asp variant in *MAP2K1* in lymphocyte development and function, the degree of the immune response impairment, as well as immuno-endocrine correlations. A comparative analysis of the symptomatology and immunological workup of two CFCS patients with hypogammaglobulinemia and our patient is displayed in Supplementary Table S2. Noteworthy, different missense variants in the same gene that lead to increased activity of the mutated protein may have distinct activating potential resulting in a variable degree of dysregulation in downstream signaling pathways. It is therefore possible that an individual immunophenotype may be ascribed to the different MAPK genotype (57). The presence of other potentially pathogenic variants that could contribute to the immunodeficiency has been excluded by careful bioinformatic analysis of the WGS data using an immunodeficiency gene panel and minor allele frequency threshold below or equal to 3%. Only two heterozygous variants, NM 000066.4:c.1282.C > T (p.Arg428Ter) in the *C8B* gene and NM 001083116.3:c.272C > T (p.Ala91Val) in the *PRF1* gene, were predicted as pathogenic. However, both variants were associated wit autosomal recessive disorders and therefore, their contribution to the immunophenotype of our patient seems unlikely. Further functional experimental and clinical studies are required for the precise delineation of the effect of both c.364 A > G; p.Asn122Asp and c.389A > G; p.Tyr130Cys missense substitutions on the MAP2K1 protein and the corresponding CFCS immunophenotype. Although the role of mutations in classical genes in components of the RAS/MAPK pathway has been elegantly studied (5, 8, 9, 58), the effect of disease-modifying altered mi-RNAs expression profiles has been revealed thereby highlighting a contribution of epigenetic regulation on *MAP2K1* and the phenotypic immuno-endocrine features in CFCS (59). It is also worth noting that genes in the RAS-MAPK pathway are among the most frequently deregulated genes in human cancer due to their regulatory role in cell proliferation, differentiation and survival. Interestingly, the genetic aberrations resulting in deregulated activation of the RAS-MAPK signaling pathway which have been recently reported in a spectrum of hematopoietic malignancies include the same N122D *MAP2K1* variant found in our CFCS patient (60). This N122D alteration occurs in the kinase domain of the *MAP2K1* gene, in the regulatory helix, while other mutations identified in CFCS are clustered.

The ever-increasing progress in genetic studies, contributing to establishing the definitive CFC diagnosis and shedding light on the interrelated genotype-phenotype heterogeneity of clinical syndromes belonging to the group of RASopathies needs to be highlighted. Herein, we add new phenotypic features of humoral immunodeficiency to the syndromic symptomatology of CFC with a novel mutation in *MAP2K1.* In this patient, multidisciplinary care of specialists in pediatric endocrinology, dermatology, cardiology, neurology, and physiotherapy is indicated, under the pediatric clinical immunologist’s supervision.

## Data Availability

The datasets for this article are not publicly available due to concerns regarding participant/patient anonymity. Requests to access the datasets should be directed to the corresponding author.

## Patient perspective

Finding a causative mutation and establishing a definitive genetic diagnosis means primarily an explanation for the multifaceted disease. Both for the family and leading physicians, it paves the way for future diagnostic and therapeutic interventions, shedding light on the expanded phenotype with immunodeficiency. Importantly, the everyday struggling with the child’s intellectual and motor disabilities, failure to thrive, recurrent infections, increased risk of tumorigenesis, frequent medical consultations and treatments with SCIg and GH, is a disease burden for the patient’s relatives. To cope with the multisystemic syndrome of CFC, medical and social support is needed for the family to enhance undertaking positive health-promoting stimulating activities. Therefore, the *de novo* nature of the pathogenic *MAP2K1* variant has an important informative role for the family.

## References

[1] Riller Q Rieux-Laucat F. RASopathies: from germline mutations to somatic and multigenic diseases. Biomed J. (2021) 44:422–32. 10.1016/j.bj.2021.06.004 34175492 PMC8514848

[2] Niemeyer C. RAS Diseases in children. Haematologica. (2014) 99:1653–62. 10.3324/haematol.2014.114595 25420281 PMC4222471

[3] Hebron KE Hernandez ER Yohe ME. The RASopathies: from pathogenetics to therapeutics. Dis Model Mech. (2022) 15:dmm049107. 1242/dmm.049107 35178568 10.1242/dmm.049107 PMC8862741

[4] Fromm Longo J Carroll SL. The RASopathies: biology, genetics and therapeutic options. Adv Cancer Res. (2022) 153:305–41. 10.1016/bs.acr.2021.07.007 35101235

[5] Rauen KA. Defining RASopathy. Dis Model Mech. (2022) 15:dmm049344. 10.1242/dmm.049344 35103797 PMC8821523

[6] Cizmarova M Kostalova L Pribilincova Z Lasabova Z Hlavata T Kovacs L Rasopathies – dysmorphic syndromes with short stature and risk of malignancy. Endocr Regul. (2013) 47:217–22. 10.4149/endo_2013_04_217 24156711

[7] Cizmarova M Hlinkova K Bertok S Kotnik P Duba HC Bertalan R New mutations associated with Rasopathies in a central European population and genotype-phenotype correlations. Ann Hum Genet. (2016) 80:50–62. 10.1111/ahg.12140 26607044

[8] Allanson JE Anneren G Aoki Y Armour CM Bondeson M Cave H Cardio-facio-cutaneous syndrome: does genotype predict phenotype? Am J Med Genet C Semina Med Genet. (2011) 157:129–35. 10.1002/ajmg.c.30295 PMC3086095 21495173

[9] Popov IK Hiat SM Whalen S Keren B Ruivenkamp C van Haeringen A A YHWAZ variant associated with cardiofaciocutaneous syndrome activates the RAF-ERK pathway. Front Physiol. (2019) 10:388. 10.3389/fphys.2019.00388 31024343 PMC6465419

[10] Serra G Felice S Antona V Di Pace MR Giuffre M Piro E Cardio-facio-cutaneous syndrome and gastrointestinal defects: report on a newborn with 19p13.3 deletion including the *MAP2K2* gene. Ital J Pediatr. (2022) 48:65. 10.1186/s13052-022-01241-6 35509048 PMC9069788

[11] Pierpont MEM Magoulas PL Adi S Kavamura MI Neri G Noonan J Cardio-facio-cutaneous syndrome: clinical features, diagnosis, and management guidelines. Pediatrics. (2014) 134:1149–62. 10.1542/peds.2013-3189 PMC4179092 25180280

[12] Rauen KA. The RASopathies. Annu Rev Genomics Hum Genet. (2013) 14:355–69. 10.1146/annurev-genom-091212-153523 23875798 PMC4115674

[13] Kavamura MI Peres CA Alchorne MMA Brunoni D. CFC index for the diagnosis of cardiofaciocutaneous syndrome. Am J Med Genet. (2002) 112:12–6. 10.1002/ajmg.10681 12239713

[14] Pierpont EI Semrud-Clikeman M Pierpont ME. Variability in clinical and neuropsychological features of individuals with *MAP2K1* mutations. Am J Med Genet A. (2017) 173:452–9. 10.1002/ajmg.a.38044 27862862

[15] Johnson B Goldberg-Strassler D Gripp K Thacker M Leoni C Stevenson D. Function and disability in children with costello syndrome and cardiofaciocutaneous syndrome. Am J Med Genet A. (2015) 167:40–4. 10.1002/ajmg.a.36828 25346259

[16] Cao H Alrejaye N Klein OD Goodwin AF Oberoi S. A review of craniofacial and dental findings in RASopathies. Orthod Craniofac Res. (2017) 20:32–8. 10.1111/ocr.12144 28643916 PMC5942188

[17] Allanson JE. Objective studies of the face of noonan, cardio-facio-cutaneous, and costello syndromes: a comparison of three disorders of the Ras/MAPK signaling pathway. Am J Med. Genet A. (2017) 170:2570–7. 10.1002/ajmg.a.37736 27155212

[18] Morice-Picard F Ezzedine K Delrue MA Arveiler B Fergelot P Taieb A Cutaneous manifestations in costello and cardiofaciocutaneous syndrome: report of 18 cases and literaturę review. Pediatr Dermatol. (2013) 30:665–73. 10.1111/j.1755-148X.2008.00511.x 24283439

[19] Urban J Qi L Zhao H Rybak I Rauen KA Kiruru M. Comparison of hair manifestations in cardio-facio-cutaneous and costello syndromes highlights the influence of the RAS pathway on hair growth. J Eur Acad Dermatol Venerol. (2020) 34:601–7. 10.1111/jdv.16082 PMC7050334 31736117

[20] Lee CL Tan LTH Lin HY Hwu WL Lee NC Chien YH Cardiac manifestations and gene mutations of patients with RASopathies in Taiwan. Am J Med Genet A. (2020) 182:357–64. 10.1002/ajmg.a.61429 31837205

[21] Ramos-Kuri M Meka SH Salamanca-Buentello F Hajjar RJ Lipskaia L Chemaly ER. Molecules linked to Ras signaling as therapeutic targets in cardiac pathologies. Biol Res. (2021) 54:23. 10.1186/s40659-021-00342-6 34344467 PMC8330049

[22] Matalon DR Stevenson DA Bhoj EJ Santani AB Keena B Cohen MS Congenital polyvalvular disease expands the cardiac phenotype of the RASopathies. Am J Med Genet A. (2021) 185:1486–93. 10.1002/ajmg.a.62146 33683002 PMC8711298

[23] Noonan JA. Noonan syndrome and related disorders: alterations in growth and puberty. Rev Endocr Metab Disord. (2006) 7:251–5. 10.1007/s11154-006-9021-1 17177115 PMC1894828

[24] Celik N Cinaz P Bideci A Yuce O Emeksiz HC Doger E Cardio-facio-cutaneous syndrome with precocious puberty, growth hormone deficiency and hyperprolactinemia. J Clin Res Pediatr Endocrinol. (2014) 8:55–8. 10.4274/Jcrpe.1151 PMC3986741 24637312

[25] Siano MA Marchetti V Pagano S Di Candia F Alessio M De Brasi D Risk of autoimmune diseases in patients with RASopathies: systematic study of humoral and cellular immunity. Orphanet J Rare Dis. (2021) 16:410. 10.1186/s13023-021-02050-6 34600590 PMC8487584

[26] Neven Q Boulanger C Bruwier A de Ville de Goyet M Meyts I Moens L Clinical spectrum of Ras-associated autoimmune leukoproliferative disorder (RALD). J Clin Immunol. (2021) 41:51–8. 10.1007/s10875-020-00883-7 33011939

[27] Kratz CP Franke L Kohlschmidt N Kazmierczak N Finckh B Bier A Cancer spectrum and frequency among children with noonan, costello, and cardio-facio-cutaneous syndromes. Br J Cancer. (2015) 112:1392–7. 10.1038/bjc.2015.75 25742478 PMC4402457

[28] Aoki Y Niihori T Narumi Y Kure S Matsubara Y. The RAS/MAPK syndromes: novel roles of the RAS pathway in human genetic disorders. Hum Mutat. (2008) 29:992–1006. 10.1002/humu.20748 18470943

[29] Ohtake A Aoki Y Saito Y Niihori T Shibuya A Kure S Non-hodgkin lymphoma in a patient with cardiofaciocutaneous syndrome. J Pediatr Hematol Oncol. (2011) 33:342–6. 10.1097/MPH.0b013e3181df5e5b 20523244

[30] Aoki Y Matsubara Y. Ras/MAPK syndromes and childhood hemato-oncological diseases. Int J Hematol. (2013) 97:30–6. 10.1007/s12185-012-1239-y 23250860

[31] Leoni C Tedesco M Onesimo R Giorgio V Rigante D Zampino G. Immunoglobulin deficiency associated with a *MAP2K1-*related mutation causing cardio-facio-cutaneous syndrome. Immunol Lett. (2020) 227:79–80. 10.1016/j.imlet.2020.08.009 32866538

[32] Sowińska-Seidler A Sztromwasser P Zawadzka K Sielski D Bukowska-Olech E Zawadzki P The first report of biallelic missense mutations in the *SFRP4* gene causing pyle disease in two siblings. Front Genet. (2020) 11:593407. 10.3389/fgene.2020.593.407 33193738 PMC7646522

[33] Kaja E Lejman A Sielski D Sypniewski M Gambin T Dawidziuk M The thousand Polish genoms – a database of Polish variant allele frequencies. Int J Mol Sci. (2022) 23:4532. 10.3390/ijms23094532 35562925 PMC9104289

[34] Kopanos C Tsiolkas V Kouris A Chapple CF Albarca Aguillera M Meyer R Varsome: the human genomic variant search engine. Bioinformatics. (2019) 35:1978–80. 10.1093/bioinformatics/bty897 30376034 PMC6546127

[35] Richards S Aziz N Bale S Bick D Das S Gastier-Foster J Standards and guidelines for the interpretation of sequence variants: a joint consensus recommendation of the American college of medical genetics and genomics and the association for molecular pathology. Genet Med. (2015) 17:405–24. 10.1038/gim.2015.30 25741868 PMC4544753

[36] Piątosa B Wolska-Kuśnierz B Pac M Siewiera K Gałkowska E Bernatowska E. B cell subsets in healthy children: reference values for evaluation of B cell maturation proces in peripheral blood. Cytometry B. (2010) 78B:372–81. 10.1002/cyto.b.20536 20533385

[37] Schatorje EJH Gemen EFA Driessen GJA Leuvenink J van Hout RWNM de Vries E. Paediatric reference values for the peripheral T cell compartment. Scand J Immunol. (2012) 75:436–44. 10.1111/j.1365-3083.2012.02671.x 22420532

[38] Ciara E Pelc M Jurkiewicz D Kugaudo M Gieruszczak-Białek D Skórka A Is diagnosing cardio-facio-cutaneous (CFC) syndrome still a challenge? Delineation of the phenotype in 15 Polish patients with proven mutations, including novel mutations in the *BRAF* gene. Eur J Med Genet. (2015) 58:14–20. 10.1016/j.ejmg.2014.11.002 25463315

[39] Myers A Bernstein JA Brennan ML Curry C Esplin ED Fisher J Perinatal features of the RASopathies: noonan syndrome, cardiofaciocutaneous syndrome and costello syndrome. Am J Med Genet. (2014) 9999:1–8. 10.1002/ajmg.a.36737 25250515

[40] Tajan M Paccoud R Branka S Edouard T Yart A. The rasopathy family: consequences of germline activation of the RAS/MAPK pathway. Endocr Rev. (2018) 39:676–700. 10.1210/er.2017-00232 29924299

[41] Dard L Bellance N Lacombe D Rossignol R. RAS Signalling in energy metabolism and rare human diseases. Biochim Biophys Acta Bioenerg. (2018) 1859:845–67. 10.1016/j.bbabio.2018.05.003 29750912

[42] Aftab S Dattani MT. Pathogenesis of growth failure in RASopathies. Pediatr Endocrinol Rev. (2019) 16:447–58. 10.17458/per.vol16.2019.ad.pathogenesisrasopathies 31115196

[43] Tamburrino F Gibertoni D Rossi C Scarano E Perri A Montanari F Response to long-term growth hormone therapy in patients affected by RASopathies and growth hormone deficiency: patterns of growth, puberty and final height data. Am J Med Genet. (2015) 167:2786–94. 10.1002/ajmg.a.37260 26227443

[44] Kosztyła-Hojna B Borys J Zdrojkowski M Duchnowska E Kraszewska A Wasilewska D Phoniatric, audiological, orodental and speech problems in a boy with cardio-facio-cutaneous syndrome type 3 (CFC 3) due to a pathogenic variant in *MAP2K1*- case study. Appl Clin Genet. (2021) 14:389–98. 10.2147/TACG.S316215 34522120 PMC8433288

[45] Tajan M Pernin-Grandjean J Beton N Gennero I Capilla F Neel BG Noonan syndrome-causing SHP2 mutants impair ERK-dependent chondrocyte differentiation during endochondral bone growth. Hum Mol Genet. (2018) 27:2276–89. 10.1093/hmg/ddy133 29659837 PMC6005060

[46] Zeitler P Sirivardana G. Stimulation of mitogen-activated protein kinase pathway in rat somatotrophs by growth hormone-releasing hormone. Endocrine. (2000) 12:257–64. 10.1385/ENDO:12:3:257 10963046

[47] Rodriguez F Gaete X Cassorla F. Etiology and treatment of growth delay in noonan syndrome. Front Endocrinol. (2021) 12:691240. 10.3389/fendo.2021.691240 PMC8212989 34149626

[48] Cantrell D. Signaling in lymphocyte activation. Cold Spring Harbor Perspect Biol. (2015) 7:a018788. 10.1101/cshperspect.a018788 PMC4448613 26032717

[49] Kortum RL Sommers CL Pinski JM Alexander CP Merrill RK Li W Deconstructing Ras signaling in the thymus. Mol Cell Biol. (2012) 32:2748–59. 10.1128/MCB.00317-12 22586275 PMC3416180

[50] Kortum RL Roquette-Jazdanian AK Samelson LE. Ras and extracellular signal-regulated kinase signaling in thymocytes and T cells. Trends Immunol. (2013) 34:259–68. 10.1016/j.it.2013.02.004 23506953 PMC3856398

[51] Priatel JJ Chen X Dhanji S Abraham N Teh H. RasGRP1 transmits prodifferentiation TCR signaling that is crucial for CD4 T cell development. J Immunol. (2006) 177:1470–80. 10.4049/jimmunol.177.3.1470 16849453

[52] Janas ML Turner M. Interaction of ras with p110γ is required for thymic beta-selection in the mouse. J Immunol. (2012) 187:4667–75. 10.4049/jimmunol.1101949 PMC3198841 21930962

[53] Redmond MT Scherzer R Prince BT. Novel genetic discoveries in primary immunodeficiency disorders. Clin Rev Allergy Immunol. (2022) 63(1):55–74. 10.1007/s12016-021-08881-2 35020168 PMC8753955

[54] MacIaurin JD Weiner OD. Multiple sources of original amplification within the B-cell RAS/MAPK pathway. Mol Biol Cell. (2019) 30:1610–20. 10.1091/mbc.E18-09-0560 31042097 PMC6727637

[55] Riegel K Rajalingam K. The non-linearity of RAF-MEK signaling in dendritic cells. Cell Cycle. (2020) 18:2249–59. 10.1080/15384101.2020.1795990 PMC7513879 32752922

[56] Chen J Che L Xu C Zhao S Yang J Li M Cardio-facio-cutaneous syndrome-associated pathogenic MAP2K1 variants activate autophagy. Gene. (2020) 733:144369. 10.1016/j.gene.2020.144369 31972311

[57] Bromberg-White JL Andersen NJ Duesbery NS. MEK Genomics in development and disease. Brief Funct Genomics. (2012) 11:300–10. 10.1093/bfgp/els022 22753777 PMC3398258

[58] Aoki Y Niihori T Inoue SI Matsubara Y. Recent advances in RASopathies. J Hum Genet. (2016) 61:33–9. 10.1038/jhg.2015.114 26446362

[59] Biso de Carvalho J Loss de Morais G dos Santos Vieira TC Chaves Rabelo N Clinton Llerena J de Carvalho Gonzalez SM miRNA genetic variants alter their secondary structure and expression in patients with RASopathies syndromes. Front Genet. (2019) 10:1144. 10.3389/fgene.2019.01144 31798637 PMC6863982

[60] Vendramini E Bomben R Pozzo F Bittolo T Tissino E Gattei V KRAS And RAS-MAPK pathway deregulation in mature B cel lymphoproliferative disorders. Cancers. (2022) 14:666. 10.3390/cancers14030666 35158933 PMC8833570

