# Supplementary material for: Case report: The cardio-facio-cutaneous syndrome due to a novel germline mutation in MAP2K1: A multifaceted disease with immunodeficiency and short stature
Source: Front Pediatr. 2022 Oct 14;10:990111. doi: 10.3389/fped.2022.990111 (PMC9614356; doi:10.3389/fped.2022.990111)
Supplement: Supplementary file 1 [file Table1.docx]

Supplementary Table 1. The overview and the pathogenicity prediction of c.364A>G; p.Asn122Asp variant found in the *MAP2K1* gene.

| **Nomenclature** | **HGVS transcript** | LRG_725:c.[364A>G];[364=] |
| --- | --- | --- |
|  | **HGVS protein** | NP_002746.1:p.[(Asn122Asp)];[(122=)] |
|  | **HGVS gDNA** | Chr15(GRCh38):g.66436818A>G |
|  | **dbSNP** | rs876657651 |
| **Classification** | **ACMG**  **PP5 Very strong**  **PM1 Strong**  **PM2 Strong**  **PS2 Strong** | Pathogenic |
|  | **ClinVar** | Pathogenic |
| **Frequencies** | **gnomAD exomes** | not found |
|  | **gnomAD genomes** | not found |
| **Pathogenicity scores** | **BayesDel addAF (dbNSFP v4.2)** | Damaging (score: 0.09717) |
|  | **BayesDel noAF (dbNSFP v4.2)** | Tolerated (score: -0.0982) |
|  | **CADD v1.6** | Deleterious (score: 27) |
|  | **DEOGEN2 (dbNSFP v4.2)** | Tolerated (score: 0.4428) |
|  | **EIGEN (dbNSFP v4.2)** | Uncertain (score: 0.3172) |
|  | **EIGEN PC (dbNSFP v4.2)** | Pathogenic (score: 0.4334) |
|  | **FATHMM (dbNSFP v4.2)** | Damaging (score: -3.18) |
|  | **FATHMM-MKL (dbNSFP v4.2)** | Damaging (score: 0.9906) |
|  | **FATHMM-XF (dbNSFP v4.2)** | Damaging (score: 0.9387) |
|  | **LIST-S2 (dbNSFP v4.2)** | Damaging (score: 0.9579) |
|  | **LRT (dbNSFP v4.2)** | Deleterious (score: 0) |
|  | **M-CAP (dbNSFP v4.2)** | Damaging (score: 0.1782) |
|  | **SIFT (v6.2.0)** | Deleterious (score: 0) |
|  | **MVP (dbNSFP v4.20)** | Benign (score: 0.8106) |
|  | **MutPred (dbNSFP v4.2)** | Benign (score: 0.409) |
|  | **Mutation assessor (dbNSFP v4.2)** | Neutral (score: 0.155) |
|  | **MutationTaster (dbNSFP v4.2)** | Disease causing (accuracy: 1) |
|  | **PROVEAN (dbNSFP v4.2)** | Damaging (score: --4.44) |
|  | **Polyphen2 HDIV (dbNSFP v4.2)** | Probably damaging (score: 0.885) |
|  | **Polyphen2 HVAR (dbNSFP v4.2)** | Probably damaging (score: 0.665) |
|  | **PrimateAI (dbNSFP v4.2)** | Damaging (score: 0.84) |
|  | **SIFT (dbNSFP v4.2)** | Tolerated (score: 0.077) |
|  | **SIFT4G (dbNSFP v4.2)** | Tolerated (score: 0.163) |
| **Conservation Scores** | **PhastCons100way (dbNSFP v4.2)** | score: 1 |
|  | **PhyloP100way (dbNSFP v4.2)** | score: 9.189 |
|  | **PhyloP17way (dbNSFP v4.2)** | score: 0.7559 |
|  | **PhyloP30way (dbNSFP v4.2)** | score: 1.3079 |
|  | **PhastCons17way (dbNSFP v4.2)** | score: 0.9969 |
|  | **PhastCons30way (dbNSFP v4.2)** | score: 1 |
|  | **fitCons-gm (dbNSFP v4.2)** | score: 0.7248 |
|  | **fitCons H1 (H1-hESC) (dbNSFP v4.2)** | score: 0.7248 |
|  | **fitCons HU (HUVEC) (dbNSFP v4.2)** | score: 0.7144 |
|  | **Integrated fitCons (dbNSFP v4.2)** | score:0.7065 |
|  | **SiPhy29way (dbNSFP v4.2)** | score: 15.0077 |
|  | **MPC (dbNSFP v4.2)** | score: 2.6332 |
|  | **bstatistic (dbNSFP v4.2)** | value: 866 |

Note: Online software used: **Varsome Premium**. ACMG criteria: **PP5 Very strong** - using strength “Very Strong” because ClinVar classifies this variant as Pathogenic (expert panel, 5 submissions), associated with Cardiofaciocutaneous Syndrome 3, Female Pseudo-Turner Syndrome, Noonan Spectrum Disorder and 7 more; **PM1 Strong** - using strength “Strong” because hot-spot of length 17 amino-acids has 23 missense/in-frame variants (19 pathogenic variants and 4 uncertain variants), which qualifies as a dense hot-spot; **PM2 Strong** - using strength “Strong” because the position is strongly conserved (phyloP100way = 9.19 is greater than 7.2), variant not found in gnomAD exomes (unable to check gnomAD exomes coverage), variant not found in gnomAD genomes (with good gnomAD genomes coverage = 31.4); **PS2 Strong** - variant is confirmed *de novo* (both maternity and paternity confirmed) in a patient with the disease and no family history.
