# Supplementary material for: Case report: The cardio-facio-cutaneous syndrome due to a novel germline mutation in MAP2K1: A multifaceted disease with immunodeficiency and short stature
Source: Front Pediatr. 2022 Oct 14;10:990111. doi: 10.3389/fped.2022.990111 (PMC9614356; doi:10.3389/fped.2022.990111)
Supplement: Supplementary file 2 [file Table2.docx]

Supplementary Table 2. Comparative clinical analysis of two CFCS patients with the same MAP2K1 variant and immunodeficiency with our patient with the novel MAP2K1 variant

| **CLINICAL DATA** | Patient #1 | Patient #2 | Patient #3 |
| --- | --- | --- | --- |
| Reference | Leoni C et al. (14) | Leoni C et al. (14) | Szczawińska-Popłonyk et al. (Current report) |
| MAP2K1 variant | c.389A>G; p.Tyr130Cys | c.389A>G; p.Tyr130Cys | c.364A>G; p.Asn122Asp |
| Age at report | 21 | 3 | 7 |
| Symptomatology | Craniofacial features  Growth retardation  Intellectual disability  Seizures  Mitral and tricuspid regurgitation  Bilateral hip dyslocation | Craniofacial features  Growth retardation  Intellectual disability  Patent foramen ovale | Craniofacial features  Growth retardation  Intellectual disability  Severe hypotrophy  Pulmonary valvular stenosis  Malformation of ventricles and corpus callosum |
| Immunodeficiency | No severe infections  No lymphadenopathy  No hepatosplenomegaly | Mild upper airways infections  No severe infections  No lymphadenopathy  No hepatosplenomegaly | Recurrent severe respiratory infections  Urinary tract infections  Sepsis  Recurrent fevers  No lymphadenopathy  No hepatosplenomegaly |
| Immunological workup | Low IgG, IgM, and IgA levels  Low IgG1, IgG2, IgG3, and IgG4 subclasses  Low switched memory B cells | Low IgM and IgA  Impaired antigen-specific response to diphtheria and tetanus toxoids | Low IgG, IgM, and IgA levels  Low IgG1,IgG3, and IgG4 subclasses  Low switched memory B cells and plasmablasts  Impaired antigen-specific response to diphtheria and tetanus toxoids  Low T CD3+, T CD4+ helper relative counts, decreased T cell thymic output (RTE), and  low T CD4+/T CD8+ ratio |
